# Front-of-Neck Access Cannot Overcome Distal Tracheobronchial Obstruction: A Fatal Case of Mochi Impaction

**DOI:** 10.7759/cureus.110406

**Published:** 2026-06-07

**Authors:** Kentaro Nagata, Taku Mayahara, Eisaku Maruo, Tomohiro Katayama

**Affiliations:** 1 Department of Emergency and General Medicine, Kobe Ekisaikai Hospital, Kobe, JPN

**Keywords:** airway obstruction, cricothyroidotomy, foreign body aspiration, front-of-neck access, mochi, ventilation failure

## Abstract

Emergency front-of-neck access (FONA) is a last-resort intervention in "cannot intubate, cannot oxygenate" situations. The underlying assumption is that establishing an airway at the cricothyroid membrane will restore ventilation. We report a fatal case in which FONA via percutaneous cricothyroidotomy failed to restore ventilation in a patient with cardiac arrest due to mochi (sticky rice cake) aspiration.

A 51-year-old man presented in pulseless electrical activity cardiac arrest after choking on mochi. Bag-mask ventilation was ineffective, and the oropharynx was densely packed with mochi, precluding visualization of the epiglottis. Percutaneous cricothyroidotomy was performed, but ventilation remained ineffective despite technically successful placement. Subsequent endotracheal intubation after oral clearance also failed to achieve ventilation. Postmortem computed tomography revealed a patent airway at the cricothyroid level with obstructing foreign material in the mid-trachea and near-complete occlusion at the carina. This case illustrates that FONA cannot overcome distal tracheobronchial obstruction and highlights a structural limitation of front-of-neck airway access in the management of mochi aspiration.

## Introduction

Emergency front-of-neck access (FONA) via cricothyroidotomy is recommended as the final rescue step in "cannot intubate, cannot oxygenate" (CICO) scenarios, in which both endotracheal intubation and supraglottic ventilation have failed and the patient faces imminent death from hypoxia [1,2]. The fundamental assumption underlying this intervention is that securing an airway at the cricothyroid membrane will bypass the obstruction and restore ventilation. However, this assumption holds only when the obstruction lies at or above the cricothyroid level; it fails when significant obstruction exists in the distal tracheobronchial tree, below the reach of any front-of-neck airway device.

Mochi (sticky rice cake) is a well-recognized cause of fatal airway obstruction in Japan [3,4]. Its adhesive, highly deformable properties allow it to conform to the airway lumen and migrate beyond the glottis into the tracheobronchial tree. We report a fatal case in which a prehospital mochi aspiration resulted in cardiac arrest and a secondary CICO state upon hospital arrival. Postmortem computed tomography (CT) clarified the mechanism of refractory ventilation failure following technically successful FONA, revealing mochi impacted in the distal trachea and carina.

## Case presentation

A 51-year-old man with a history of depression and atopic dermatitis was eating grilled mochi with his family when he placed two large square pieces into his mouth in succession and choked. He clutched his neck, consistent with the universal choking sign, and walked to the bathroom, where his father attempted to help him expel the mochi without success. The Heimlich maneuver was not performed. His family called emergency medical services (EMS) approximately 10 minutes after the onset of choking.

EMS arrived 7 minutes after the call (approximately 17 minutes after symptom onset) and found the patient in a seated position, unconscious, being supported from behind by his father. No pulse was palpable, and cardiopulmonary resuscitation (CPR) was initiated on scene. Cardiac monitoring revealed bradycardic pulseless electrical activity (PEA). The interval from symptom onset to initiation of CPR by EMS was approximately 17 minutes; the exact no-flow time was unknown. The patient arrived at our emergency department 30 minutes after the EMS call (approximately 40 minutes after symptom onset).

On arrival, the patient remained in cardiac arrest with bradycardic PEA. His pupils were bilaterally dilated at 6 mm without light reflex. CPR was continued, and intravenous access was established. Administration of adrenaline 1 mg was initiated and repeated at four-minute intervals thereafter. Bag-mask ventilation produced no chest rise. Direct laryngoscopy revealed the oral cavity densely packed with mochi; the epiglottis could not be visualized. Removal was attempted using manual extraction and Magill forceps, but the mochi was highly viscous and adherent to the mucosal surfaces, and only small fragments could be removed at a time.

Given the inability to secure a definitive airway through the oral route, the decision was made to proceed with FONA. Percutaneous cricothyroidotomy was performed using a Mini-Trach II device (Portex, Williams Medical Supplies, Rhymney, South Wales; 4-mm internal diameter). The device was successfully placed 12 minutes after hospital arrival. Ventilation was attempted using a bag-valve device with high pressures, but chest rise remained absent.

Oral clearance of mochi using laryngoscopy and Magill forceps was temporarily interrupted during the cricothyroidotomy procedure and was resumed after placement of the Mini-Trach II. Despite continued attempts, removal remained difficult. Twenty minutes after arrival, the cardiac rhythm deteriorated to asystole. By 29 minutes after arrival, oral clearance was finally completed, the Mini-Trach II was removed, and endotracheal intubation was performed. A transport ventilator (ParaPAC) was connected with a pressure-relief valve set at 40 cmH₂O; however, airway pressures consistently exceeded this threshold when attempting to deliver a tidal volume of 300 mL, and chest rise was not achieved. End-tidal CO₂ measured immediately after intubation was 14 mmHg. Resuscitative efforts were ultimately unsuccessful; return of spontaneous circulation (ROSC) was not achieved at any point. Death was confirmed 50 minutes after hospital arrival.

**Figure 1:**
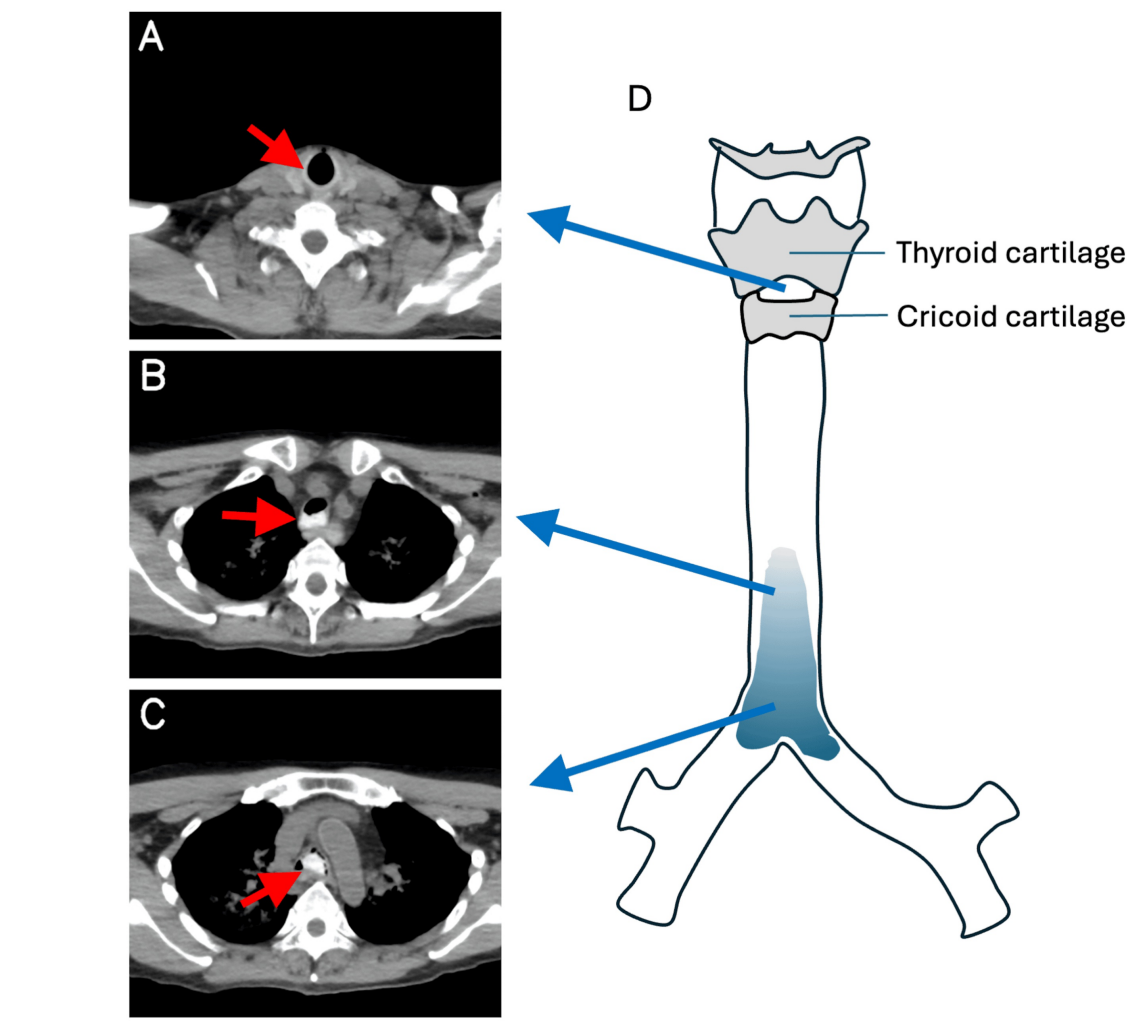
Postmortem axial computed tomography Postmortem axial computed tomography (5-mm slice thickness; mediastinal window, width 350 HU, level 30 HU) demonstrating distal tracheal obstruction due to mochi impaction. (A) Patent airway at the level of the cricothyroid membrane (red arrow). (B) High-attenuation foreign material within the mid-tracheal lumen (red arrow); the tracheal air column is partially obliterated by the impacted material. (C) Near-complete obstruction at the carina (red arrow). (D) Schematic representation of the airway demonstrating the relationship between the cricothyroidotomy site and the levels of mochi impaction; shaded areas indicate the distribution of impacted material, progressing from partial obstruction at the mid-trachea to near-complete occlusion at the carina. Blue arrows indicate the axial CT levels corresponding to panels A, B, and C. Only axial images were available for review; multiplanar reconstructions could not be obtained.

Postmortem axial CT demonstrated a patent airway at the level of the cricothyroid membrane (Figure 1A), high-attenuation foreign material (approximately 198 HU) within the mid-tracheal lumen extending over eight consecutive slices (approximately 40 mm in craniocaudal length) (Figure 1B), and near-complete obstruction at the carina (Figure 1C). These findings indicated that ventilation failure resulted from obstruction distal to the cricothyroidotomy site and beyond the airway access achieved by subsequent endotracheal intubation. Autopsy was not performed, as consent from the family could not be obtained.

In retrospect, three clinical features distinguished this case from routine supraglottic foreign body obstruction: (1) the mochi was highly viscous and adherent to the mucosa, precluding en bloc removal; (2) ventilation failed despite technically successful percutaneous cricothyroidotomy; and (3) ventilation remained impossible even after complete oral clearance and standard endotracheal intubation. This triad should alert clinicians to the possibility of foreign body impaction distal to the cricothyroid level.

## Discussion

FONA is a cornerstone of difficult airway management in CICO situations. However, it is effective only when the obstruction lies above, or at the level bypassed by, the cricothyroidotomy. This case demonstrates a structural limitation of this rescue strategy: when critical obstruction lies distal to the cricothyroid level, FONA cannot restore ventilation regardless of technical success. Postmortem CT revealed obstruction extending from the mid-trachea to near-complete occlusion at the carina, while the airway at the cricothyroid level remained patent. The subsequent failure of standard endotracheal intubation to achieve ventilation further supported distal tracheobronchial obstruction as the main mechanism of ventilation failure.

The choice of the Mini-Trach II device warrants comment. The Mini-Trach II has a narrow 4-mm internal diameter and is not equivalent to a standard cuffed surgical cricothyrotomy tube for rescue ventilation; a standard surgical cricothyrotomy with a larger-bore tube would generally be preferred [5]. The narrow diameter likely contributed to inadequate ventilation through the cricothyroidotomy and precluded passage of a standard adult bronchoscope (which typically requires an internal diameter of ≥5 mm), thereby eliminating any possibility of endoscopic assessment or intervention through the front-of-neck access site. However, because ventilation also failed after endotracheal intubation with a standard tube, the poor outcome cannot be attributed solely to device selection; even a standard surgical cricothyrotomy tube would likely not have overcome the distal tracheobronchial obstruction demonstrated on postmortem imaging.

The physical properties of mochi are central to understanding the pattern of obstruction seen in this case. Mochi is highly adhesive and deformable, allowing it to conform to the contour of the airway lumen and resist removal by suction or forceps [4]. Because its physical properties are temperature-dependent, heated mochi may be particularly soft and adhesive at the time of aspiration, facilitating impaction within the airway [6]. These properties may allow mochi to migrate distally beyond the glottis into the tracheobronchial tree, producing a pattern of obstruction that cannot be relieved by airway access above the level of impaction. Distal migration of aspirated material may occur before or during attempted airway management. In the present case, the interval between symptom onset and definitive airway management may have allowed distal migration of mochi, although the timing of tracheobronchial impaction cannot be determined.

Several techniques have been described for managing distal tracheobronchial foreign body obstruction once endotracheal intubation is achieved. Deliberate advancement of the endotracheal tube into a mainstem bronchus to achieve single-lung ventilation may bypass a carinal or proximal bronchial obstruction [7]. Vacuum extraction using a meconium aspirator connected to an endotracheal tube has been reported as a successful technique for tracheal foreign body removal [8]. Bronchoscopic retrieval using a cryoprobe has also been described specifically for mochi impaction, exploiting the temperature-dependent viscosity of mochi: rapid cooling hardens the material and facilitates en bloc removal [9]. However, none of these techniques were attempted in the present case. The oral cavity was completely obstructed, and endotracheal intubation could not be achieved until 29 minutes after hospital arrival, by which time the patient had already been in asystole for 9 minutes. In retrospective review, deliberate endobronchial intubation was identified as the most feasible intervention had earlier airway access been possible, although its effectiveness against adhesive, deformable material, such as mochi, remains uncertain.

## Conclusions

This case illustrates a fundamental limitation of FONA: it cannot overcome obstruction distal to the cricothyroid membrane. Although the narrow diameter of the percutaneous cricothyroidotomy device may have compounded the ventilatory failure, the persistence of refractory ventilation even after standard endotracheal intubation supports distal tracheobronchial obstruction as the principal mechanism. FONA remains an essential and potentially life-saving rescue strategy when obstruction is at or above the glottic level; the limitation described here applies specifically to cases in which foreign material has migrated into the distal tracheobronchial tree. When ventilation fails despite technically successful front-of-neck airway access, distal tracheobronchial obstruction should be considered. The adhesive, deformable nature of mochi may facilitate migration below the glottis and conformity to the airway lumen, potentially producing a pattern of obstruction for which conventional airway rescue strategies are insufficient. In the absence of autopsy confirmation, these inferences are based on postmortem imaging findings.
